# Enhanced Morphological Characterization of Cellulose Nano/Microfibers through Image Skeleton Analysis

**DOI:** 10.3390/nano11082077

**Published:** 2021-08-16

**Authors:** Jose Luis Sanchez-Salvador, Cristina Campano, Patricio Lopez-Exposito, Quim Tarrés, Pere Mutjé, Marc Delgado-Aguilar, M. Concepcion Monte, Angeles Blanco

**Affiliations:** 1 Department of Chemical Engineering and Materials, Facultad de Ciencias Químicas, Universidad Complutense de Madrid, Avenida Complutense, 28040 Madrid, Spain; josanc03@ucm.es (J.L.S.-S.); ccampano@ucm.es (C.C.); cmonte@ucm.es (M.C.M.); 2 Departamento de Bioingeniería e Ingeniería Aeroespacial, Universidad Carlos III de Madrid, Avda. de la Universidad 30, 28911 Leganés, Spain; palopeze@pa.uc3m.es; 3 Group LEPAMAP, Department of Chemical Engineering, University of Girona, C/M. Aurèlia Campmany 61, 17071 Girona, Spain; joaquimagusti.tarres@udg.edu (Q.T.); pere.mutje@udg.edu (P.M.); m.delgado@udg.edu (M.D.-A.)

**Keywords:** nanocellulose, morphology, cellulose nanofibers, gel point, microscopy, image skeleton analysis, quality control

## Abstract

The present paper proposes a novel approach for the morphological characterization of cellulose nano and microfibers suspensions (CMF/CNFs) based on the analysis of eroded CMF/CNF microscopy images. This approach offers a detailed morphological characterization and quantification of the micro and nanofibers networks present in the product, which allows the mode of fibrillation associated to the different CMF/CNF extraction conditions to be discerned. This information is needed to control CMF/CNF quality during industrial production. Five cellulose raw materials, from wood and non-wood sources, were subjected to mechanical, enzymatic, and (2,2,6,6-Tetramethylpiperidin-1-yl)oxyl (TEMPO)-mediated oxidative pre-treatments followed by different homogenization sequences to obtain products of different morphologies. Skeleton analysis of microscopy images provided in-depth morphological information of CMF/CNFs that, complemented with aspect ratio information, estimated from gel point data, allowed the quantification of: (i) fibers peeling after mechanical pretreatment; (ii) fibers shortening induced by enzymes, and (iii) CMF/CNF entanglement from TEMPO-mediated oxidation. Being mostly based on optical microscopy and image analysis, the present method is easy to implement at industrial scale as a tool to monitor and control CMF/CNF quality and homogeneity.

## 1. Introduction

Cellulose micro and nanofibers suspensions (CMF/CNFs) are produced from a wide variety of sources such as wood, non-wood plants, grasses, marine animals, algae, fungi, invertebrates, and bacteria [1,2]. Although the most common sources to produce CMF/CNFs at large scale are hardwood and softwood virgin pulps [3,4], other pulps from agricultural wastes and non-wood plants are being increasingly considered in view of circular economy concepts or their availability in factories for local markets. In comparison to wood, non-wood plants and crops residues generally present lower proportions of lignin and hemicellulose, which yields a cellulose content close to 70%. Therefore, the isolation of cellulose fibers and the fibrillation processes are also less energy intensive [5]. Among non-wood plants, sisal, jute, and hemp have the potential to become commercially interesting sources of cellulose [6]. Although it is possible to find some studies in the literature aiming to isolate either CNFs or cellulose nanocrystals (CNCs) from these non-wood plants [7,8], a more profound knowledge is needed to facilitate their use.

The mechanical methods used to obtain CMF/CNFs involve intensive disintegration processes to produce the separation of nanofibrils. These treatments entail high energy consumption and result in a limited nanofibrillation efficiency [9]. Therefore, chemical, enzymatic, mechanical, or combined pretreatments are used to facilitate the fibrillation process [1,10]. (2,2,6,6-Tetramethylpiperidin-1-yl)oxyl (TEMPO)-mediated oxidation is one of the most common chemical pretreatments, producing a high nanofibrillation degree due to the production of interfibrillar repulsive forces between the fibrils via the conversion of primary hydroxyl groups present in cellulose into carboxylate groups [11]. Mechanical refining increases the branching of the fiber via mechanical shearing [12]. Although this pretreatment has been proved to ease the subsequent fibrillation process, the product obtained is highly heterogeneous [13]. Finally, enzymatic hydrolysis is an environmental friendly process that does not generate toxic residues and produce the breakdown of cellulose polymer into smaller polymer branches favoring the posterior mechanical treatment [14]. In this study, in order to refer to the different range of products, obtained with the several pretreatments and homogenization passes, i.e., branched pulp microfibers (several tens of micrometer in diameter), exfoliated microfibrils (several tens to hundreds of nanometers in diameter), and nanofibrils (several nanometers in diameter and several micrometers in length), we have used the acronym CMF/CNFs.

It is well known that the pretreatment will determine the final CMF/CNF properties, especially with respect to morphology [1]. Although some studies have addressed the morphological differences induced by different pretreatments [15,16,17], their assessment has been only qualitative, using diameter, length, or aspect ratio distributions, and performed on model cellulose sources, i.e., bleached hardwood or softwood Kraft pulps. Apart from qualitative assessment, the description of morphology using the size parameters could be unreliable and meaningful, since CMF/CNFs need to be described in the network state they will be in when they are applied. Therefore, there is a need for simple, quantitative methods to characterize the morphology of CMF/CNFs in such a way that one could control the homogeneity of the products and, on the other hand, grasp the effect these products would have in the final application depending on its quality [18].

Skeletonization is an image processing technique based on object erosion of wide use in fields such as character recognition and the analysis of biomedical images [19]. The application of skeletonization to image objects results in another object which is an abstraction of the first. The skeleton object contains both shape and topological structures of the original object, but since these features are simplified down to lines and nodes, the transformed object can be more easily analyzed. Skeletonization has been used, for example, to measure directly and quantitatively the branching of carbon-black aggregates from transmission electron microscopy (TEM) images [20] or to assess the shape of KCl crystals during their formation [21].

The novelty of this work is the quantitative assessment of fibrillation by measuring the nodes in the skeletonized fibers detected on images acquired through optical microscopy (OM) and TEM when necessary. This technique can provide relevant information of the branching, peeling, and breakage of the fibers during the treatments. These data can be complemented with the aspect ratio of CMF/CNFs easily estimated through the gel point (GP) methodology [22]. To validate this hypothesis, five CMF/CNF products have been characterized obtained from wood (eucalyptus and pine) and non-wood plants (sisal, jute, and hemp). Three pretreatments (TEMPO-mediated oxidation, enzymatic hydrolysis, and refining) and several cycles of homogenization were used to evaluate their effect on CMF/CNF morphology. The five samples have been deeply characterized using traditional methods, the results of which are compared with the proposed procedure.

## 2. Materials and Methods

### 2.1. Materials

Five bleached raw materials were considered for CMF/CNF production: kraft softwood pulp (pine), kindly provided by Arauco (Arauco, Chile); kraft hardwood pulp (eucalyptus) from ENCE (Navia, Spain); jute, hemp and sisal bleached kraft pulps were supplied by Celesa (Tortosa, Spain). The reagents used for TEMPO-mediated oxidation were 10% *w*/*v* NaClO, TEMPO reagent and NaBr (Merck, Madrid, Spain). NaOH, HCl, NaCl, and H_2_SO_4_ were supplied by Merck (Madrid, Spain), crystal violet by Sigma (Madrid, Spain), and Poly-L-Lysine solution by Electron Microscopy Sciences (Hatfield, PA, USA). 

### 2.2. Pretreatments of Cellulose

The raw materials were left to soak in water for 24 h to favor fiber swelling. Then, they were disintegrated in a PTI pulp disintegrator (Vorchdorf, Austria) at 90,000 revolutions and 1.5 wt.% consistency.

Refining, used as mechanical pretreatment, was performed in a PFI mill manufactured by Hamjem Maskin AS (Hamar, Norway). The pulp consistency was adjusted to 10 wt.% for 20,000 revolutions.

For the enzymatic hydrolysis, the cellulose suspension consistency was set at 5 wt.% and acidified with 0.1 M HCl until pH 5. The suspension was heated until 50 °C under constant stirring. At constant pH and temperature, the enzyme solution of Novozym 476 (Novozymes A/S, Kalundborg, Denmark) was dropped into the suspension using two different doses, 80 (E80) and 240 mg/g of pulp (E240). The enzyme solution contains 2% of cellulases and its activity factor is 4500 CNF-CA/g cellulose (tested over a CMC substrate). The suspension was maintained for 4 h under stirring conditions. Finally, enzymatic hydrolysis was stopped by increasing the temperature up to 80 °C for 30 min.

The third pretreatment was TEMPO-mediated oxidation, performed in a glass reactor at 1 wt.% cellulose consistency with 0.1 mmol TEMPO/g pulp and 1 mmol NaBr/g pulp [11]. Then, 5 (T5) or 15 mmol NaClO/g pulp (T15) were added to start the reaction, which was conducted at room temperature and stirring conditions. During the process, the pH was controlled to 10, adding a 2 M NaOH solution dropwise. The reaction was considered to end when the pH remained constant with no need to add additional NaOH.

### 2.3. Production of Cellulose Nanofibers

Pretreated pulps were mechanically fibrillated using a high-pressure laboratory homogenizer NS1001L PANDA 2K-GEA (GEA Niro Soavy, Parma, Italy) to produce CMF/CNFs of different nanofibrillation yield from each raw material. The consistency of the pulp was adjusted to 1 wt.%. To achieve different nanofibrillation yields, five pressure sequences (PS) were carried out. From less intensive to more intensive high-pressure homogenization (HPH), the PS are described below:

- PS1:3 passes of HPH at 300 bars.
- PS2:3 passes of HPH at 300 bars and then 1 pass at 600 bars.
- PS3:3 passes of HPH at 300 bars and 3 additional passes at 600 bars.
- PS4:3 passes of HPH at 300 bars, 3 passes at 600 bars, and then 1 pass at 900 bars.
- PS5:3 passes of HPH at 300 bars, 3 passes at 600 bars, and 3 passes at 900 bars.

### 2.4. Characterization of Raw Materials and CNFs

Extractives, Klason and soluble lignin, cellulose, hemicellulose, and ash were measured in the raw materials. Extractives of the samples were determined via Soxhlet extraction according to TAPPI T204. Total lignin, cellulose, and hemicellulose content of raw materials were obtained following NREL/TP-510-42618. First, 300 mg of cellulose was hydrolyzed with 3 mL 72 wt.% H_2_SO_4_ for 1 h in a water bath at 30 °C. Then, 84 g of deionized water was added and introduced in an autoclave for one hour at 121 °C. The hydrolyzed samples were vacuum filtered. Klason lignin remained in the filter whereas the soluble lignin fraction was obtained by measuring the absorbance of the filtrate in the UV-visible spectrophotometer. Hemicellulose and cellulose content were analyzed via high performance liquid chromatography (HPLC) from the filtrate after neutralization with calcium carbonate and passed through a 0.2 µm filter. Finally, the ash content was determined via calcination, according to TAPPI T211.

The crystallinity index (CrI) of raw materials was obtained via X-ray diffraction (XRD) spectra using a Philips X’Pert MPD X-Ray diffractometer (Malvern Panalytical, Malvern, UK) with an auto-divergent slit fitted with a graphite monochromator, Cu-Kα radiation, and operated at 45 kV and 40 mA. XRD patterns were recorded in a range from 3 to 80° at a scanning speed of 1.5°/min. Segal’s method was used to determine CrI [23].

The carboxyl content of the pretreated fibers and the cationic demand (CD) of the obtained CMF/CNFs were determined using conductometric titration and colloidal titration, respectively, according to Delgado-Aguilar et al. (2015) [24].

### 2.5. Aspect Ratio: Gel Point Methodology

Aspect ratio was obtained using simplified GP methodology based on the sedimentation of the fibers. Traditionally, the GP was calculated from the derivative at the origin of the curve initial concentration (Ø*_o_*) vs. the ratio of sediment height (*H_s_*) to initial suspension height (*H_o_*) as indicated in Equation (1) [22]. A simplification of the *GP* was obtained using increments of the derivative at the origin of the curve Ø*_o_* vs. *H_s_*/*H_o_* (Equation (1)) [25].

(1) GP=limHsHo→0d∅odHsHo≈∅oi − ∅o0HsHoi − HsHo0=∅oiHsHoi 

To determine the *GP*, a CMF/CNF suspension was prepared using deionized water and stirred for 10 min. During the agitation, 200 µL of crystal violet 0.1 wt.% was added to favor sediment visualization [9], and 250 mL of the suspension was left to settle in a graduated cylinder until the sediment reached a steady value that indicated the complete deposition of fibers. The selection of the initial concentration was performed in order to obtain a sediment height around 4–12% of the total height, due to the difficulty found in measuring the height accurately at lower sedimentation values. On the other hand, a high sedimentation height would cause the increment of concentrations that appears in Equation (2), for which the derivative has been substituted, and moves away from the limit of Hs/Ho of close to zero.

In this study, the initial concentration was varied from 0.01 to 0.1 wt.% except in the case of CNFs pretreated through TEMPO-mediated oxidation whose sedimentation concentration was from 0.1 to 0.3 wt.% of dry CNFs.

The aspect ratio was calculated from the GP values, given in kg/m^3^, according to Varanasi et al. (2013) [22], assuming a density of fibers around 1500 kg/m^3^ and using the crowding number theory described by Martinez et al. (2001) [26] with Equation (2).

Aspect ratio = 6.0 × (GP/1000)^−0.5^ (2)

### 2.6. Imaging

The morphology of CMF/CNFs was characterized via OM and TEM. Fibers were visualized via OM using a Zeiss Axio Lab.A1 OM and a Zeiss AxioCam ERc 5s color microscope camera (Carl Zeiss Microscopy GmbH, Göttingen, Germany) under 5× magnification. TEM analyses were carried out at the National Centre of Electronic Microscopy (Madrid, Spain) with a JEM 1400 microscope from JEOL (Tokyo, Japan). To prepare the samples, 15 µL of 10% poly-L-lysine solution was added on a copper grid covered with a Formvar/carbon continuous layer. Then, 12 µL of 0.005% (*w*/*w*) CMF/CNF suspension was deposited and left to dry before analysis [27].

OM and TEM images were processed and analyzed to retrieve morphological information. In view of the large number of images, processing and analysis were automatized through the implementation and application of various software scripts.

First, images were binarized using Fiji, a distribution of ImageJ, an open-source image processing software package. OM images were pre-processed to enhance contrast and highlight fiber structures through the application of a minimum filter. Then, they were binarized and further processed to remove holes corresponding to translucent sections within the fibers. TEM images were first bandpass filtered and their background was subtracted. Subsequently, an auto-threshold was applied followed by minimum filtering. The images were then binarized.

Both OM and TEM images were segmented and the particles detected analyzed in terms of size and shape and skeletonized using the script published previously for characterizing cellulose nanocrystals morphology [28]. The number of nodes and branches were determined for each image skeleton obtained. This process was also automatized. An example of this procedure has been provided as supporting information in Figure S1.

## 3. Results and Discussion

### 3.1. Characterization of Raw Materials

Table 1 summarizes the components characterization of the raw materials. The compositions of sisal and jute were similar to the one of eucalyptus, having around 80% cellulose and 15% hemicellulose (in the form of xylose) as major components. On the other hand, hemp and pine presented very similar compositions, with cellulose and hemicellulose proportions around 90% and 7% (also xylose), respectively. These results are in accordance with Pickering et al. (2016), Pappu et al. (2016), and Alila et al. (2013) [29,30,31]. These similarities were also observed in the lignin content when considering the sum of Klason lignin and the lignin fraction soluble in acid. Over 90% of this value represents the soluble fraction, while the content in Klason lignin is almost negligible in all cases. Regarding crystallinity, as expected, the crystalline regions found in pine were more abundant than in eucalyptus, whereas the crystallinities of the three non-wood plant material were in the range 76–78%, close to that of pine, as it has been previously observed [31,32].

The different features of all five fibrous materials can be observed in the microscopy images included in Table 1. The characteristic short and bundled fibers of eucalyptus and the long and crystalline ones of pine acknowledged in the literature [33] can be observed in the images. Despite both wood sources having been treated through the Kraft process, there is a clear contrast between the levels of deterioration in the fibers. While eucalyptus fibers showed a smooth surface, the presence of ramifications and entanglement is ostensible in the pine fibers. The main reason for this lies in the greater propensity for degradation of softwood pulps mainly associated to their morphology, composition, and crystallinity [34]. With regards to the non-wood samples, sisal and jute fibers showed a smooth surface, as in the eucalyptus according to Mwaikambo et al. (2002) [32], although in these two cases fiber lengths were visibly much longer. Hemp fibers exhibited a ramified aspect that resembled those of pine to some extent [35].

### 3.2. Morphological Changes of Fibers during Micro/Nanofibers Production

#### 3.2.1. Mechanical Pretreatment

**Figure 1:**
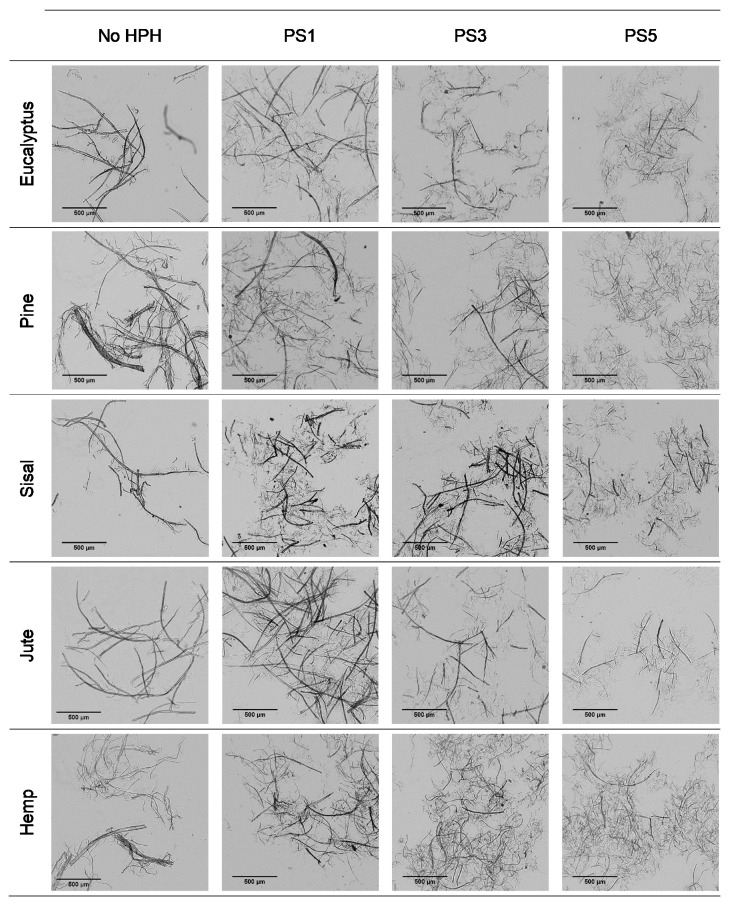
Optical microscopy images at 5× magnification of pretreated cellulose and CMF/CNFs with the mechanical pretreatment.

Figure 1 shows the modification in the fiber morphology with the mechanical pretreatment through OM images. The main morphological change in cellulose fibers expected from the mechanical refining is the separation of microfibrils from the primary structure, which could result in either more ramified fibers or individual microfibers coming from the complete peeling of primary fibers.

It is observed that eucalyptus fibers remained almost unaltered after pretreatment, which produced only a few branches around the fibers. However, an intensive defibrillation was observed with more microfibrils around the cellulose backbones after homogenization with the formation of networks. Although fibrillation was further increased with the severity of the PS, the fibers did not reach a homogeneous diameter and two different types of fibers were observed: some with a higher length and more straight morphology, being likely the product of the initial fibers peeling, and some microfibrils with a diameter of a few microns and a bundled appearance, either completely separated or protruding from the masts.

**Figure 2:**
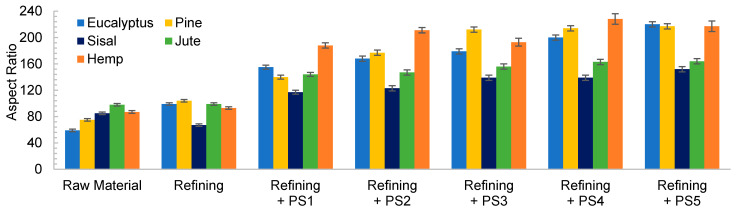
Evolution of aspect ratio when a refining pretreatment and different pressure sequences are used to produce CMF/CNFs.

The low initial aspect ratio of eucalyptus (Figure 2), around 60, significantly increased after refining and subsequent HPH intensities as result of a decrease in the diameter of the original fibers due to peeling. The aspect ratio was subsequently increased with the severity of the HPH, reaching a value of around 220 in PS5 because of a predominant fibrillation effect. Sisal and jute showed a similar fibrillation effect as in eucalyptus, as observed in Figure 1 and Figure 2, whereby the shortening of the primary fibers proceeded at the same time that the samples were fibrillated, maintaining the average aspect ratio of the sample. This fact could be associated to a higher content of hemicellulose in these three sources, which would apply a strong holding of fiber bundles [36].

In the case of pine and hemp, given that the raw fibers were already highly ramified, the effect of refining was not as appreciable as in the other cases. In this case, OM images show only a slight increase in fibrillation after pretreatment. However, when HPH was applied, the number of branches in cellulose backbones and the microfibrils were totally separated at PS1 and PS3. In contrast to eucalyptus, a low distribution range of fiber diameters was reached after PS5. This observation was in accordance with the values of the aspect ratio, which showed a more pronounced increase with the homogenization of up to 215 and 220 for pine and hemp, respectively. This fact could be associated to the best fibrillation of the cellulose backbones with mechanical shearing, which has been explained through the easier deterioration that softwood fibers typically suffer because of their higher rigidity.

Despite morphological images of fibers treated with PS3 and PS5 exhibiting a huge difference of size, they reach a maximum aspect ratio that remains almost constant with further homogenization. During homogenization at the highest pressures, not only are microfibrils separated, but also shortened, resulting in a similar aspect ratio.

#### 3.2.2. Enzymatic Hydrolysis

Table S1 shows an increase in the CD with the severity of the pressure sequence in HPH and also with the increase in the enzyme dosage, indicating an increasing fibrillation level achieved through enzymatic hydrolysis. Compared to the CMF/CNFs produced with the mechanical pretreatment, CD was slightly higher, as observed previously [24]. The number of carboxyl groups (Table S2) does not vary with pretreatment, as occurs with refining, with low values around 40–50 μeq/g, as expected since no oxidation treatment was applied.

**Figure 3:**
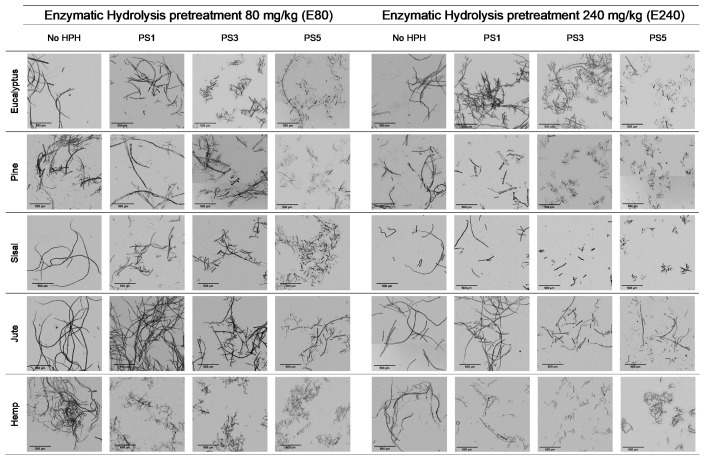
Optical microscopy images at 5× magnification of cellulose and CMF/CNFs with enzymatic hydrolysis pretreatment.

The enzymatic hydrolyzed eucalyptus and the produced CMF/CNFs showed a reduced length in the fibers compared to the initial sample in Table 1, and this effect was intensified with the increase of the enzyme dose (Figure 3). Despite the breakage of fibers being more apparent after a soft homogenization, this fact is on account of the enzymes that act in the amorphous regions of cellulose, cutting the cellulose chains and reducing their length.

**Figure 4:**
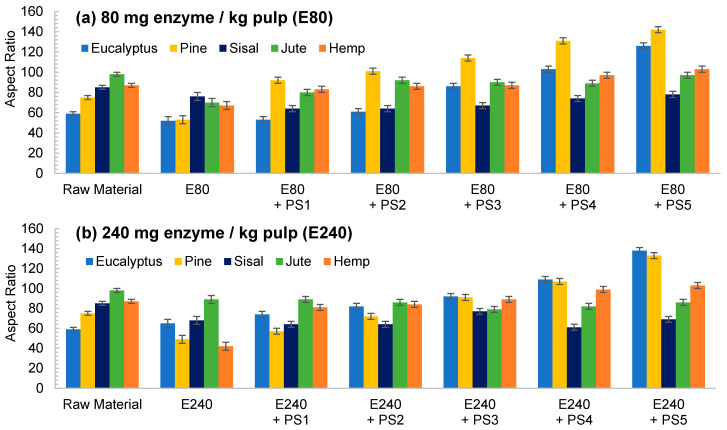
Evolution of aspect ratio in CMF/CNFs with enzymatic hydrolysis as pretreatment with different enzyme dose: (**a**) 80 mg/kg (E80); (**b**) 240 mg/kg (E240).

The aspect ratio of the E80 pretreated eucalyptus and after PS1 decreases in Figure 4a, reflecting the break of fibers. However, in E240 the effect of fibrillation counteracted the break of the fibers producing a slight increase in the aspect ratio (Figure 4b). On the other hand, a more intense homogenization produced the fibrillation of the samples at both enzyme dosages, although a very heterogeneous sample is obtained despite the high intensity of PS5. Nevertheless, the size range is more homogeneous than in the CMF/CNFs pretreated through refining.

In contrast to eucalyptus, the break of the cellulose chains of pine was already observed in the images of the pretreated non-homogenized sample. The fact that pine presents a higher crystallinity to the rest of the materials and lower hemicellulose content may suppose that the enzymes could have hydrolyzed most of the amorphous cellulose, leaving apart just the crystalline regions, with a straight morphology and a lower possibility of interaction among them. This effect was even more pronounced after homogenization, which facilitated the peeling of the pretreated fibers up to a point so that it could be identified as a type of microcrystal. This fiber breakage with a posterior defibrillation was also identified through the determination of the aspect ratio, showing a decrease after the pretreatment and then a progressive increase, as seen in Figure 4.

Although sisal and jute had a composition and morphology similar to eucalyptus, their behavior was quite different. In both samples, the number of breaks induced from the enzymatic treatment likely prevailed over the fibrillation. This was observed not only in the OM images of Figure 3, but also in Figure 4, in which the aspect ratio was slightly reduced after the pretreatment and then increases weakly after PS1, maintaining the ratio during homogenization.

The evolution of the aspect ratio with the pretreatment at the two doses of enzymes considered for the hemp was similar to those observed for pine. However, after HPH the evolution was completely different from pine with an aspect ratio almost invariable after PS1. In addition, it is probable that the break of the fibers caused by the action of the enzymes could be happening at an early stage, while the homogenization could be triggering the fibrillation of the sample, forming networks of a similar area but higher proportion of nodes and with a similar aspect ratio.

#### 3.2.3. TEMPO-Mediated Oxidation

The third pretreatment considered in this study was the TEMPO-mediated oxidation at two different dosages of oxidant: 5 mmol (T5) and 15 mmol of NaClO/g pulp (T15). With the TEMPO-mediated oxidation process, primary hydroxyl groups present in C6 of cellulose oxidize to carboxyl groups, which triggers an electrostatic repulsion between fibrils [11]. This oxidation occurs preferentially in the amorphous regions of cellulose, since they are more accessible, while the available hydroxyl groups of the crystalline edges would remain probably unaltered until the disordered regions have been completely oxidized [37]. Thus, it is expected that both crystallinity and morphology of raw material play an important role in the morphology of the CMF/CNFs.

Table S2 shows an increase in the number of carboxyl groups after the pretreatment, reaching around 820 mmol/g in eucalyptus, pine, and hemp and around 750 mmol/g in jute and sisal for T5. The slightly lower number of carboxyl groups in jute and sisal could be assigned to the higher hemicellulose content of the samples as well as crystallinity, reducing the number of amorphous areas that are accessible for oxidation. Nevertheless, the carboxyl content in T15 was similar in all samples, around 1360–1390 mmol/g, except in jute where there was a lower content, around 1300 mmol/g. At this dosage, most of the C6 positions may have been oxidized and a proportion of the more oxidized and amorphous sample could have remained in dissolved or colloidal form [38]. As showed in Table S1, the values of CD in CMF/CNFs increased gradually with the severity of the homogenization for all sources. Some studies have recently proposed the value of CD as a monitoring parameter of fibrillation degree, since it provides the proportion of surface anionic charge that is released when fibrils are split [39]. Hence, the higher values of CD obtained in CNFs produced with TEMPO-mediated oxidation pretreatment indicate they have a higher surface area than CMF/CNFs obtained with the other two types of pretreatments. It is worth mentioning that for comparison reasons, the carboxyl content, which increases considerably due to the oxidation of hydroxyl groups in C6 with the reaction catalyzed by TEMPO, has been subtracted from CD [40].

**Figure 5:**
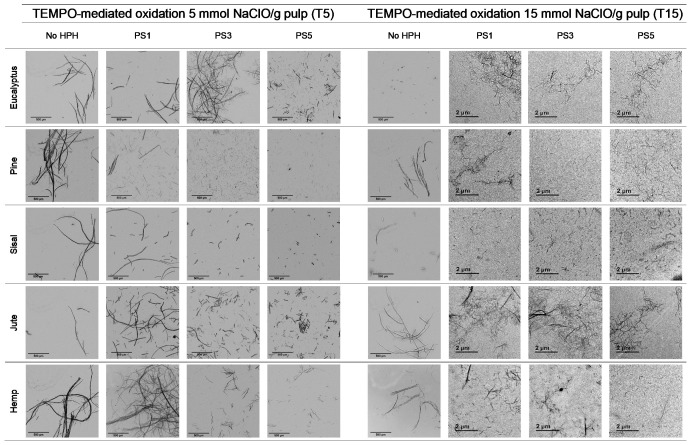
Optical microscopy images at 5× magnification and transmission electron images (TEM) of pretreated cellulose and CNFs with the TEMPO-mediated oxidation pretreatment.

Figure 5 shows OM images of pulps and CNFs after TEMPO-mediated oxidation. Considering the huge decrement in size of the CNFs accounted for by the T15 treatment, TEM images of CNFs after homogenization were considered instead of OM and the corresponding OM images are included in Figure S2.

The fibers were fragmented in shorter chains at the same time that the diameter was further reduced due to the action of the oxidant, which oxidized hydroxyl groups of C6 of amorphous regions to carboxyl groups, with higher size and anionic charge. If this treatment is very intensive, as in the case of T15, the electrostatic repulsion could not only cause the separation between the fibrils, but also their breakage in the proximities of the crystalline shaft, which would present a higher resistance to being defibrillated [41].

**Figure 6:**
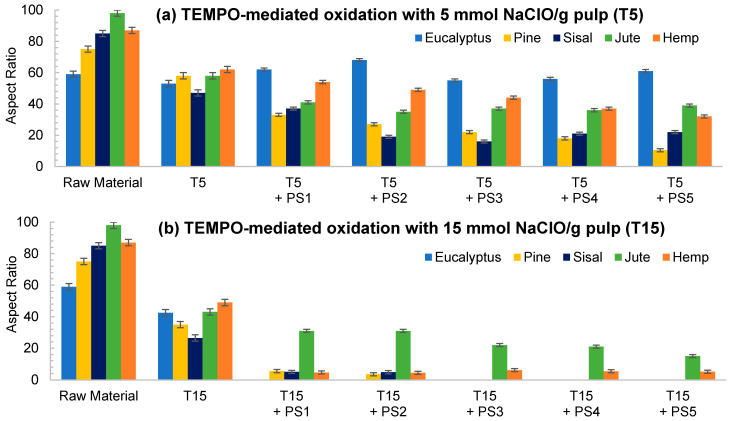
Evolution of aspect ratio in CNFs with TEMPO-mediated oxidation pretreatment. (**a**) TEMPO-mediated oxidation with 5 mmol NaClO/g pulp (T5); (**b**) TEMPO-mediated oxidation with 15 mmol NaClO/g pulp (T15).

As reflected in aspect ratio values, the split of the fibers was predominant over their shortening in most of the cellulose sources, detected via a decrease in this parameter (Figure 6). Moreover, this behavior was intensified when the oxidant dosage was increased up to T15.

TEMPO-mediated oxidation of pine produced a higher shortening effect than the one observed for eucalyptus at both oxidant doses. The presence of more crystalline regions in pine, as Table 1 indicates, may have reduced the attack of the oxidant. However, after homogenization the repulsion between the carboxyl groups produced the separation of fibrils at the same time as their shortening. After PS5, it was possible to obtain a homogeneous diameter distribution of pine which varied in scale with the oxidant dose. Nevertheless, the eucalypt sample pretreated with T5 at PS5 showed the remaining presence of some microfibers together with some other nanofibers, whereas the total fibrillation of eucalyptus CNFs was achieved with T15 with severities over the PS3, forming networks of nanofibrils, as previously described.

The aspect ratio of eucalyptus CNFs produced with T5 is slightly reduced from the raw material, and after HPH the aspect ratio does not decrease as much as other raw materials. Despite decreasing the diameter of the CNFs, the length decreases in a similar proportion forming networks, and not in a greater extent as in T15, in which the excessive oxidation shortens the CNF length in a greater proportion than the diameter.

As for pine, CNFs produced with T5 decreased progressively during homogenization until values around 10 in PS5 were reached. This low length/diameter ratio was even reduced more pronouncedly with T15, reaching values below 5 from the PS2 homogenization. After that pressure sequence it was not possible to measure the aspect ratio due to the fact that the particles found in suspension were stabilized and they did not sediment. The images in PS5 showed a rigid structure with a diameter range from 20 to 40 nm, which would indicate that they are CNC [17]. The case of pine was not unique in inducing the formation of CNC; this was also the case for sisal and hemp, in which this effect was even intensified.

Although the behavior of sisal was mostly similar to that of eucalyptus in refining and enzymatic hydrolysis, the extreme oxidation of this cellulose source after the use of T15 caused the complete breakage of the fibers. The initial morphology that sisal presents, with elongated but highly compact fibers, could have induced this behavior.

The case of jute was similar to the one of eucalyptus, in which the oxidation produced a low reduction in the shortening of the fibers compared to the other materials. With T5, as high an intensity sequence as PS3 was required to obtain separate fibers at the same time as fibrillation of the sample. The aspect ratio of T5 decreased with the pretreatment, but it was then maintained during homogenization at around 40, with a heterogeneous size distribution after PS5. On the other hand, the breakage of fibers was easily induced with T15 even at low intensity in the homogenization, like PS1. Again, the networks described for eucalyptus were shown in jute CNFs produced with T15.

To sum up, from the traditional characterization of CNFs, especially through image analysis and aspect ratio determination, one could conclude that CMF/CNFs obtained using mechanical pretreatment have a more branched morphology, with a slight reduction in the fibers’ dimension intensified with HPH. On the other hand, hydrolysis caused by enzymes induced the characteristic shortening in the fibers’ length, with a slight reduction in diameter, but only after HPH. Finally, the oxidation caused in fibers mediated by TEMPO reduced both diameter and length drastically, giving place to the creation of networks of nanofibrils. This marked tendency will be explained in detail in the next section, through the use of skeleton analysis.

### 3.3. Description of Morphology of Cellulose Nano/Microfibers through Image Skeleton Analysis

**Figure 7:**
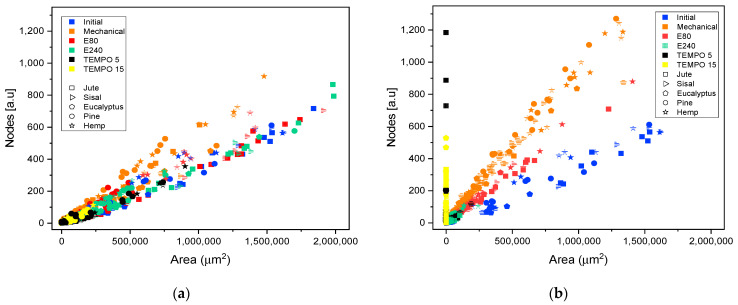
Evolution of the number of nodes quantified in the elements identified in optical microscopy images and transmission electron microscopy with their projected area, when different pretreatments are applied to produce cellulose micro and nanofibers from different sources: (**a**) after pretreatment without homogenization; (**b**) after pretreatment and HPH at PS5.

Fiber morphology was quantified in detail though the analysis of OM and TEM skeletonized images. Figure 7 depicts the effect of the different pretreatments on the ratio between the number of skeleton nodes of fibers or networks and their projected area. Firstly, in Figure 7a the effect of pretreatment was evaluated without HPH treatment, whereas Figure 7b shows the analysis performed on CMF/CNFs after the PS5 sequence. In both cases, the five raw materials were plotted with points of different geometric shapes.

In images containing fibers, CMFs, or CNFs, nodes represent two topological elements, either points in which the fiber branches or points in which fibers cross over each other due to the random deposition of fibers in the sample. Thus, the intersection of two non-ramified fibers would have one node and the area would be the result of summing the area of the two fibers. However, if those two fibers are highly branched, the ratio of the number of nodes to the same projected area of fiber would reach a much higher magnitude. It follows from this that the evolution in the number of nodes with the treatment will depend on the predominance of either fiber overlapping or the presence of branches.

For all initial samples, the relationship between number of nodes and area is linear. A higher slope can be observed in some samples as in the initial hemp, indicating a greater number of nodes for the same area associated to a greater branching of fibers, as previously indicated in the description of the raw materials.

The slope observed for the samples mechanically pretreated is higher than those of the initial samples (Figure 7a), which implies that this pretreatment favored the peeling of the fibers and had a lower impact on the length of the fibers. In the same color, Figure 7b shows an even greater increase in nodes for the same area after homogenization at PS5, relating the same behavior as previously observed in Figure 1. Moreover, the networks formed with this pretreatment can be discriminated from fibers branching using this methodology, since in both cases, the branched fibers or the networks are accounted for as single elements: increased nodes with similar area if they are simply branched or increased nodes with area reduction if we are treating with networks of micro or nanofibers.

Regarding enzymatic pretreatment, the distribution of nodes vs. projected area is maintained after pretreatment with the two doses assayed. As the OM images show (Figure 3), the morphology of the fibers was maintained after pretreatment without shortening. After PS5 with the two doses, the slopes are steeper than the raw material. In both cases, the number of nodes was increased at the expense of reducing fiber size considerably. As previously stated, enzymatic pretreatment causes a predominant reduction in the fiber length, which facilitates the consequent fibrillation using HPH. This behavior can be now explained in detail with the proposed methodology, solving the typical issues that arise from determining an average diameter or length, as well as providing a complement to the unspecific “nanofibrillation degree”.

Skeleton analysis of TEMPO-mediated oxidation pretreatment was also included in Figure 7. As in microscopy images, a scale change in CNFs obtained using this pretreatment was required. Therefore, the quantification of nodes and area in samples pretreated with T5 and homogenization at PS5 were carried out using both TEM and OM images, whereas in T15, homogenized samples were analyzed only with TEM images due to no fibers being observed in their respective OM images.

Figure 7a shows the splitting of fibers after the TEMPO-mediated oxidation pretreatment through the drastic reduction of the projected area. As occurs in enzymatic pretreatment, there is not an increase in the number of nodes after pretreatment. The reduction of the size of the fibers until obtaining highly fibrillated CNFs is consistent with the reduction in size observed in the microscopic images of Figure 5.

It is worth mentioning that, although the image technique was adapted to the scale of the CNFs, there is a gap in which the samples are not detected, either in OM or TEM, with a consequent loss of detail. This fact is observable in T5 samples with PS5 (Figure 7b) in which both microscopic techniques are useful to observe the fibrils. The results from OM images have a low slope whereas in TEM images the slope is slightly steeper. As previously indicated, the gap in which the samples are not detected would appear between both slopes, forming a kind of triangular area. A higher oxidation degree in the pretreatment, such as T15, that further reduced the size of fibers with a more homogeneous size, produces a drastic decrease in the area in which fibrils are not observable in OM images.

Taking into account Figure 7b, one could observe that the graphs of nodes vs. area could provide some kind of map in which samples subject to the same treatment are located around the same zone, with slight variations depending on the raw material.

**Figure 8:**
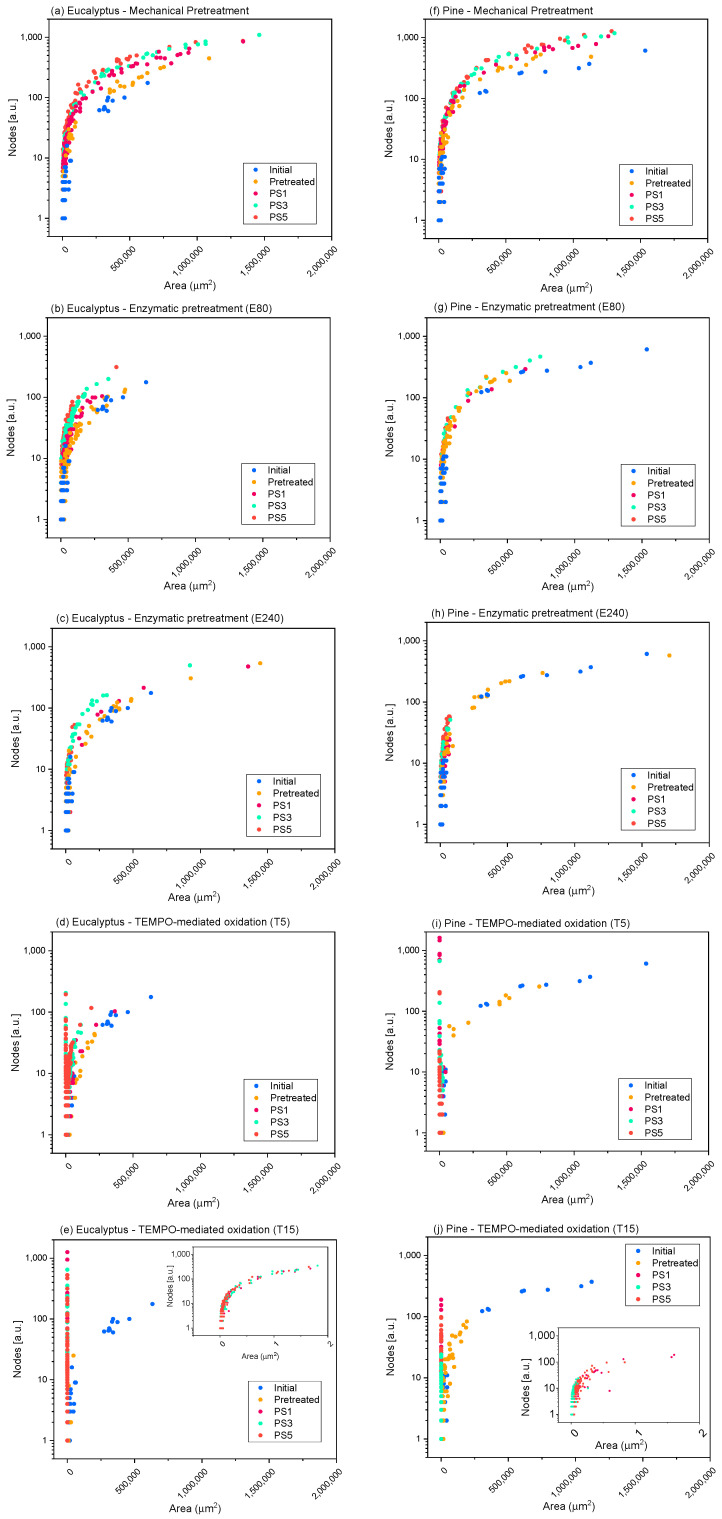
Evolution of the number of nodes quantified in the elements identified in the microscopy images with their projected area, when different pretreatments and pressure sequences are used to produce eucalypt (**a**–**e**) and pine cellulose (**f**–**j**) micro and nanofibers (CMF/CNFs). Mechanical pretreatment: (**a**,**f**); enzymatic pretreatment (E80): (**b**,**g**); enzymatic pretreatment (E240): (**c**,**h**); TEMPO-mediated oxidation (T5): (**d**,**i**); TEMPO-mediated oxidation (T15): (**e**,**j**).

Figure 8 provides an example of the methodology proposed for two of the raw materials studied, eucalyptus (Figure 8a–e) and pine (Figure 8f–j), as models. The representations of the rest of the raw materials are shown in the supplementary file (Figure S3).

In the case of pine, given that the raw fibers were already highly ramified, the effect of refining on the nodes per area ratio was not as appreciable as in eucalyptus, since a smaller increment was observed in the number of nodes, while occupying the same area, as the OM images show. The high ramification after refining is also shown in hemp (Figure S3), while for jute and sisal, not only was an increase in the number of nodes observed, but also a reduction in the area of the elements due to the separation of some microfibrils from the primary structures. Moreover, the nodes/area ratio was maintained when severity of homogenization was over PS1.

The nodes accounted for in the enzymatic pretreatment (Figure 8b,c,g,h and Figure S3) in either E80 or E240 were more similar to those determined for the initial cellulose, maintaining the same curve as the initial samples (pine, hemp) or with a very low increase in the number of nodes for the same area that was accentuated with homogenization (mainly in eucalyptus and, to a lesser extent, in jute and sisal), which could be associated to a slightly higher fibrillation of fibers than their shortening. In addition, we can observe a diminution of the particle area as the homogenization sequence is intensified, which may also have a greater number of particles without nodes (individual fibrils), indicating the higher homogeneity of these CMF/CNFs and a reduced size of fibers compared with E80.

The evolution of skeletonized samples with TEMPO-mediated oxidation pretreatment was also observed in Figure 8d,e,i,j and Figure S3. Both T5 pretreated eucalyptus and pine and their initial pulps are represented in the same curve with the same ratio nodes vs. area but with a lower proportion of large particles, which indicates the previously determined shortening of the fibers. After HPH, CNFs show a high reduction in area, while the number of nodes increases exponentially for elements that occupy the same area in respect to the raw materials. In addition, it is possible to observe the homogeneity of the samples in the curve of nodes versus area of T5. Eucalyptus or jute CNFs at PS3 and PS5 exhibit a higher area due to the presence of networks that are accounted for as one element and to the fact that larger fibers are still observable in OM images, whereas CNFs from pine showed more homogeneous CNFs, as they have been quantified only via TEM. On the other hand, the T15 pretreated samples show a more reduced area than T5 due to a more pronounced shortening of the fibers. Comparing the curves of T15-PS5 samples of eucalyptus and pine, we observe that eucalyptus shows a greater proportion of particles with a large relative area and a high number of nodes than pine, which indicates the presence of CNF networks, as Figure 5 shows. On the other hand, the reduced proportion of nodes and area in pine indicates the straight morphology typical of CNC particles.

## 4. Conclusions

Quantification of morphological features of CMF/CNFs has been successfully achieved via the analysis of eroded CMF/CNF microscopy images, which provides information on fiber morphology, ramification, and breakage. These results were complemented with aspect ratio data for a robust characterization of different CMF/CNFs, obtained through several treatments.

This novel approach allowed us to describe quantitatively the significant peeling on the fibers that is induced by the mechanical pretreatment, the shortening of fibers caused through enzymatic hydrolysis, and the formation of networks of shortened nanofibrils that is observed in CNFs after TEMPO-mediated oxidation. Thus, it was possible to differentiate between ramified fibers and networks of microfibers, through the disposition of the sample in the map nodes/area. The extent of these effects depended on the degree of crystallinity and the morphology of the cellulosic raw material.

Therefore, this work can contribute to consolidating the CMF/CNF industry, considering that a reliable characterization of CMF/CNFs is key to ensure the quality and replicability of CMF/CNF suspensions and to monitor the process, which is nowadays an industrial challenge. Thus, the approach described in this study could serve, on the one hand, as an easy and reliable method to be implemented at industrial scale for low fibrillated products and, on the other hand, for highly fibrillated products as a standard to be correlated with other methods that although do not provide enough detail in the description of the fibrillation of CMF/CNFs could be more easily implemented in-line.

## Figures and Tables

**Table 1:** Raw materials characterization: composition, crystallinity, and optical images (5× magnification).

	Eucalyptus	Pine	Sisal	Jute	Hemp
	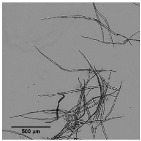	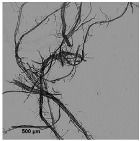	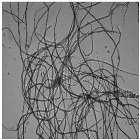	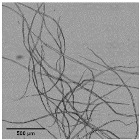	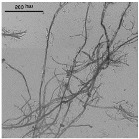
Cellulose (%)	74.6	87.7	80.7	80.2	90.0
Hemicellulose (%)	17.6	7.4	12.6	11.2	5.8
Lignin (%)	6.4	3.6	5.0	6.9	3.4
Extractives (%)	1.2	0.5	0.9	0.8	0.8
Ash (%)	0.4	0.7	0.9	0.9	0.6
CrI (%)	72.7	78.3	77.8	75.9	75.8

## Supplementary Materials

The following are available online at https://www.mdpi.com/article/10.3390/nano11082077/s1, Table S1: Cationic demand of CNFs and microfibers after homogenization; Table S2: Carboxyl content of CMF/CNFs after each pretreatment; Figure S1: Example of image skeletonization of the fibers; Figure S2: Optical Microscopy (OM) of cellulose nanofibers (CNF) pretreated with TEMPO-mediated oxidation and 15 mmol of NaClO/g pulp and different homogenization sequences; Figure S3: Evolution of the number of nodes quantified in the elements identified in the microscopy images with their projected area, when different pretreatments and pressure sequences are used to produce sisal, jute, and hemp cellulose micro and nanofibers (CMF/CNFs).
