# Supplementary material for: Enhanced Morphological Characterization of Cellulose Nano/Microfibers through Image Skeleton Analysis
Source: Nanomaterials (Basel). 2021 Aug 16;11(8):2077. doi: 10.3390/nano11082077 (PMC8398699; doi:10.3390/nano11082077)
Supplement: Supplementary file 1 [file nanomaterials-11-02077-s001.zip › nanomaterials-1313547-supplementary.pdf]

Article

Enhanced Morphological Characterization of Cellulose Nano/Microfibers through Image Skeleton Analysis

Jose Luis Sanchez-Salvador [1], Cristina Campano [1], Patricio Lopez-Exposito [2], Quim Tarrés [3], Pere Mutjé [3], Marc Delgado-Aguilar [3], M. Concepcion Monte [1] and Angeles Blanco [1,*]

[1] Department of Chemical Engineering and Materials, Complutense University of Madrid. Avda. Complutense s/n, 28040 Madrid, Spain; josanc03@ucm.es (J.L.S.-S.); ccampano@ucm.es (C.C.); cmonte@ucm.es (M.C.M.)

[2] Departamento de Bioingeniería e ingeniería Aeroespacial, Universidad Carlos III de Madrid, Avda. de la Universidad 30, 28911 Leganés, Spain; palopeze@pa.uc3m.es

[3] Group LEPAMAP, Department of Chemical Engineering, University of Girona, C/M. Aurèlia Campmany 61, 17071 Girona, Spain; joaquimagusti.tarres@udg.edu (Q.T.); pere.mutje@udg.edu (P.M.); m.delgado@udg.edu (M.D.-A.)

* Correspondence: ablanco@ucm.es; Tel.: +34-913-944-247

Table S1. Cationic demand of CNFs and microfibers after homogenization.

		**Eucalyptus (µeq/g)**	**Pine (µeq/g)**	**Sisal (µeq/g)**	**Jute (µeq/g)**	**Hemp (µeq/g)**
Refining	PS1	184	154	131	155	134
	PS3	208	188	163	182	168
	PS5	215	210	199	209	205
E80	PS1	204	181	168	186	151
	PS3	216	203	194	208	184
	PS5	232	213	214	220	210
E240	PS1	200	192	176	204	162
	PS3	224	206	203	223	200
	PS5	250	219	226	233	221
T5	PS1	1023	1009	925	995	938
	PS3	1188	1119	1035	1068	1078
	PS5	1277	1201	1098	1135	1088
T15	PS1	1594	1598	1614	1541	1599
	PS3	2012	1774	1863	1808	1801
	PS5	2059	1909	2005	2014	1991

Table S2. Carboxyl content of CNFs and microfibers after each pretreatment.

	Eucalyptus (μeq/g)	Pine (μeq/g)	Sisal (μeq/g)	Jute (μeq/g)	Hemp (μeq/g)
Refining	44	53	42	47	54
Enzymatic Hydrolysis 80 mg enzyme / kg pulp	45	53	42	47	54
Enzymatic Hydrolysis 240 mg enzyme / kg pulp	45	53	42	47	54
TEMPO-mediated oxidation 5 mmol NaClO / g pulp	821	816	742	758	821
TEMPO-mediated oxidation 15 mmol NaClO / g pulp	1385	1374	1371	1295	1363

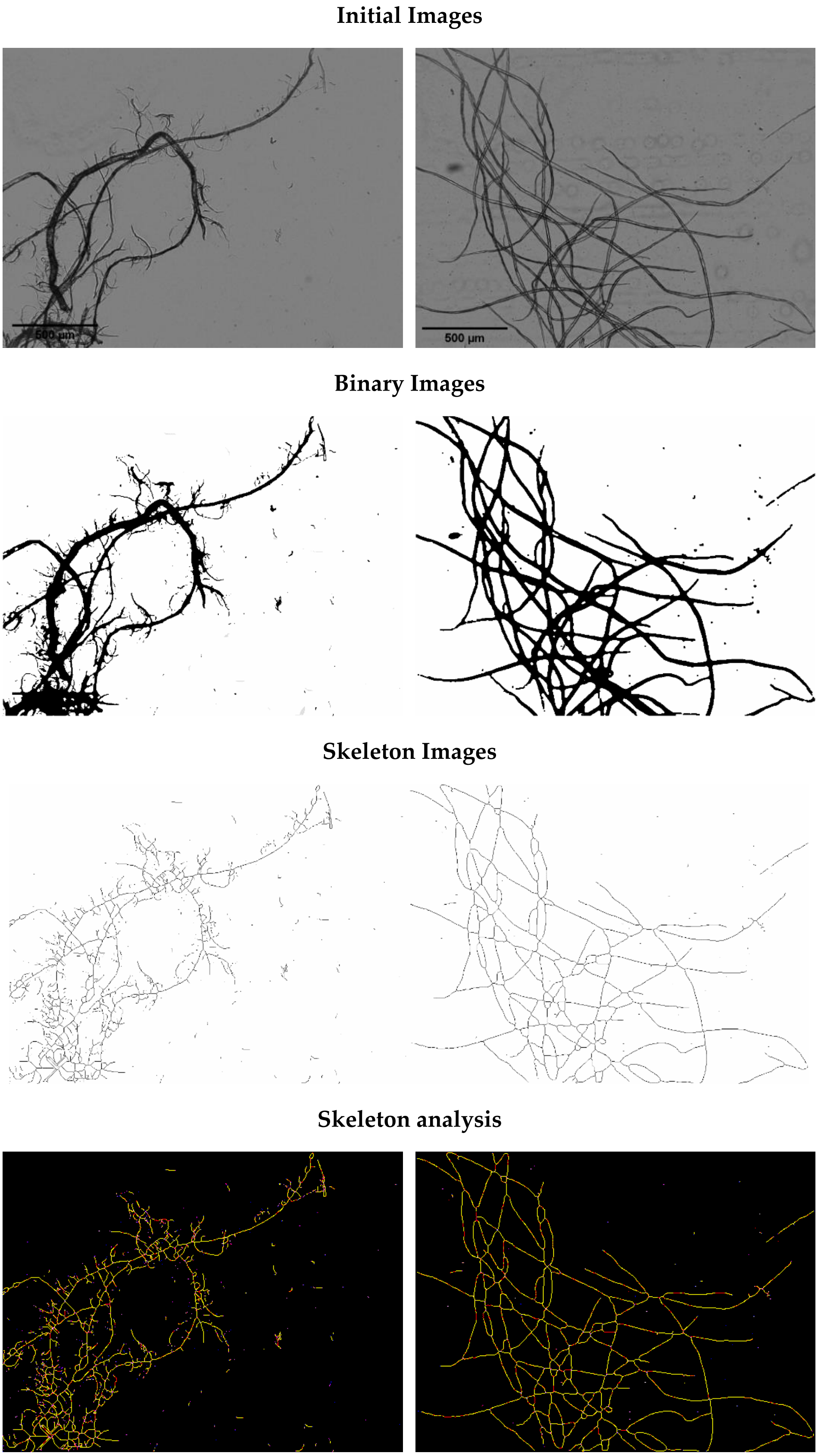

Nodes 413	Nodes 187
Branches 761	Branches 305

Figure S1. Example of image skeletonization of the fibers.

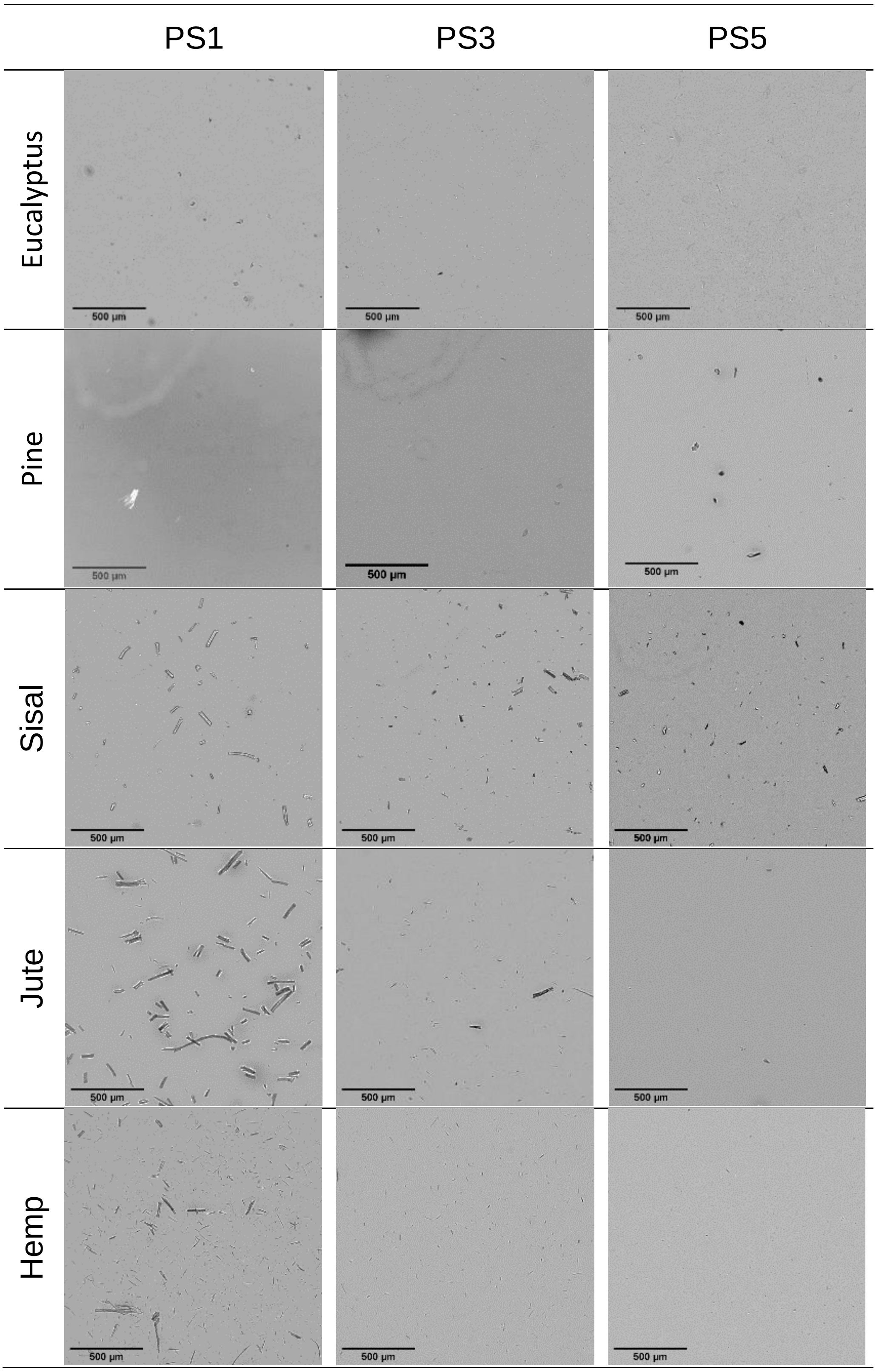

Figure S2. Optical Microscopy (OM) of cellulose nanofibers (CNF) pretreated with TEMPO-mediated oxidation and 15 mmol of NaClO/g pulp and different homogenization sequences.

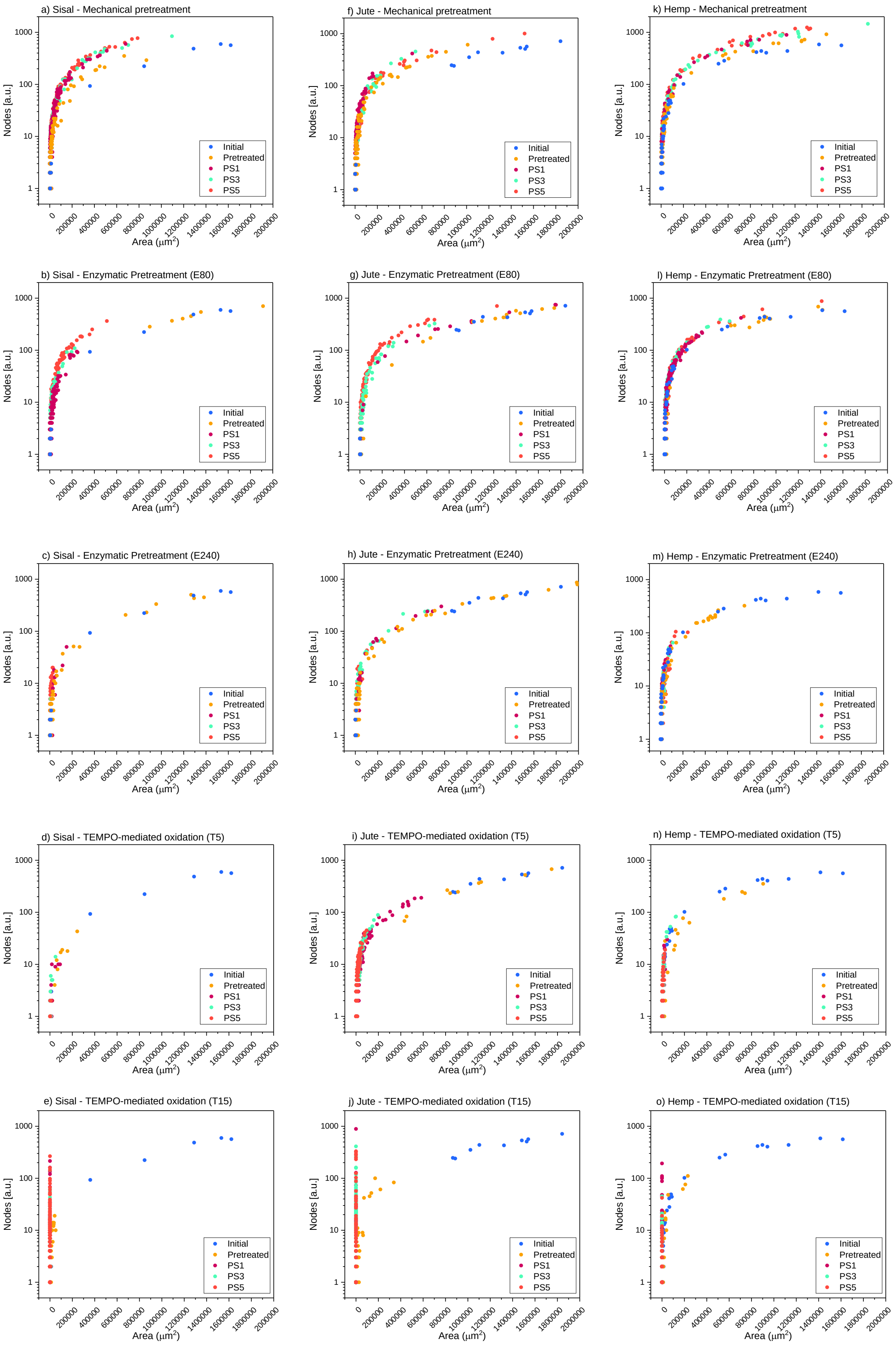

Figure S3. Evolution of the number of nodes quantified in the elements identified in the microscopy images with their projected area, when different pretreatments and pressure sequences are used to produce sisal, jute and hemp cellulose nanofibers.
